# Molecular tumour boards and molecular diagnostics for patients with cancer in the Netherlands: experiences, challenges, and aspirations

**DOI:** 10.1038/s41416-019-0489-3

**Published:** 2019-05-27

**Authors:** Annelieke E. C. A. B. Willemsen, Sarah Krausz, Marjolijn J. L. Ligtenberg, Katrien Grünberg, Harry J. M. Groen, Emile E. Voest, Edwin P. J. G. Cuppen, Hanneke W. M. van Laarhoven, Carla M. L. van Herpen

**Affiliations:** 1 Department of Medical Oncology, Radboud university medical center, Nijmegen, The Netherlands; 2 Department of Medical Oncology, Cancer Center Amsterdam, Amsterdam University Medical Center, University of Amsterdam, Amsterdam, The Netherlands; 3 Department of Pathology, Radboud university medical center, Nijmegen, The Netherlands; 4 Department of Human Genetics, Radboud university medical center, Nijmegen, The Netherlands; 5 Department of Pulmonary Diseases, University Medical Center Groningen, Groningen, The Netherlands; 6 Division of Molecular Oncology, Netherlands Cancer Institute, Amsterdam, The Netherlands; 7 Center for Molecular Medicine and Oncode Institute, University Medical Center Utrecht, Utrecht, The Netherlands; 8 Hartwig Medical Foundation, Amsterdam, The Netherlands

**Keywords:** Molecular medicine, Cancer genomics, Targeted therapies

## Abstract

Advances in molecular tumour diagnostics and the number of targeted therapies increase rapidly. Molecular tumour boards (MTBs) are designated to interpret these data and provide clinical recommendations. Not all patients with cancer have access to advice of an MTB. We aimed to determine the current status, opportunities, and challenges of the organisation of MTBs in the Netherlands. We interviewed several stakeholders about their experiences with an MTB, using template analysis. Most clinicians and patient representatives underscore the significance of an MTB, because it can stimulate rational treatment options, enrolment in clinical trials, and interdisciplinary knowledge transfer. Health insurance companies and financial managers are concerned about increasing costs. Registries to assess the clinical benefit of MTBs, guidelines on quality control, financial agreements, and logistical resources are lacking. The national organisation of MTBs and a registry of molecular and clinical data are important issues to address.

## Background

Numerous new possibilities for precision medicine for patients with cancer have arisen. To aid medical specialists in the highly complex field of molecular biology and clinical care, molecular tumour boards (MTBs) discuss the clinical relevance of genetic aberrations with detailed genomic explanation and treatment options. However, a recent survey shows that the advice of an MTB is not accessible for all patients in the Netherlands and that MTBs vary greatly in composition, tasks, tools, and workflow.^1^ Here, we determine the opportunities and challenges of the organisation of MTBs in the Netherlands, from the perspectives of different stakeholders, addressing the following questions: What is the added value of an MTB? What is needed for its optimal organisation and functioning? Which financial and logistical resources are required?

## Methods

We interviewed different stakeholders in the Netherlands about their experiences with an MTB, using a qualitative approach, applying template analysis (supplementary Table 1). We included health care professionals (medical oncologists, pulmonologists, pathologists, molecular biologists, a clinical geneticist, and a surgeon), and financial managers from hospitals with and without MTB experience, medical oncologists in training, representatives from a national cancer patient federation and from health insurance companies. All respondents provided consent. A description of data collection and data analysis is provided in the supplementary material.

## Results

Demographic data of the respondents are summarised in supplementary Table 2.

### The significance of an MTB for patients and health care professionals

Most health care professionals acknowledge the value of an MTB. Four points of significance are mentioned. (1) Improvement of patients’ outcomes by providing a multidisciplinary tailored-based treatment advice. (2) Continuous interdisciplinary knowledge transfer. (3) Cost-saving by predictive diagnostics preventing ineffective therapy. (4) Facilitation of scientific output. The patient federation encourages early referral to an MTB, as this can provide personalised (extra) treatment options and improve enrolment in clinical trials.

Some medical oncologists are more sceptical about the current significance of an MTB. (1) Most of their patients are discussed with late stage disease, which can limit treatment possibilities. (2) In contrast to many lung tumours, treatment options for other solid tumours are more limited. (3) Many current medical oncologists in training have not attended an MTB yet and do not have a clear vision of the potential of an MTB. (4) Appropriate palliative care can be delayed while waiting for an MTB advice. (5) Most clinicians from non-MTB centres and medical oncologists in training experience off-label prescription based on an actionable tumour mutation as an undesirable situation. However, others regard an MTB as a suitable forum to centralise and regulate off-label prescriptions and the patient federation experiences off-label prescription as an act of bravery of their treating physician.

### The organisation and regulation of an MTB within local, regional and national networks

Interdisciplinary collaboration is regarded as crucial and participation of a clinical scientist in molecular pathology is considered indispensable. In many MTBs, the treating clinician of the patient who is discussed is absent and relevant clinical information can be insufficient. Some MTB-centres favour more regional collaboration in order to increase the patient number of their MTB, while other MTB-centres with extensive regional collaboration do not have time for further expansion.

For the future of MTBs, some respondents propose to maintain tumour non-selective MTBs in every specialised cancer centre and design only one central MTB in the Netherlands for high-complex cases. Alternatively, molecular expertise may be integrated more within the existing tumour-specific oncology boards.

All respondents agree that evaluating the quality of an MTB is crucial to gain maximal clinical benefit. The following items warrant regulation and evaluation: (1) the goals of an MTB, including which patients will be referred, (2) the composition of an MTB, (3) registration of clinical outcomes, (4) type, quality, and validation of molecular diagnostic procedures used.

### Financial and logistical resources

Financial managers in the hospitals and representatives of health insurance companies are concerned about increasing costs of diagnostics and medication and stress the importance of evidence for the benefits of MTBs. Respondents did not express need for financial compensation for the time spent on the MTB. In community hospitals prescription of medication off-label often is not possible due to financial constraints. Health insurance companies are willing to reimburse (part of) the costs of molecular diagnostics and treatment, provided that certain conditions are satisfied: (1) criteria for referral to an MTB, (2) insight in the clinical outcomes, and (3) adherence to diagnostic quality demands and the composition of the MTB. Furthermore, in their opinion, pharmaceutical companies should contribute to reimbursement, as they benefit from an increase in the prescription of therapeutic agents. Ultimately, decisions on finances should be made on a national political level.

Logistical support is required for coordination of the MTB, facilities, and reports. A simple and widely accessible registration procedure is important. Most respondents believe an MTB report should have a description of the molecular method, a conclusion of the data with a treatment advice, and reference to supporting scientific literature. Some physicians from non-MTB centres prefer to attend the MTB to discuss their patient through teleconsulting, while others prefer referral of the patient. Clinicians feel the urge to discuss possible genetic unsolicited findings prior to the application of molecular diagnostics but differ in preferences on the details of this procedure.

The most important points of tension as described are summarised in Table 1.

**Table 1:** Points of tension

Points of tension
I. Significance of MTB for patients, health care professionals, and stakeholders
a. Early versus late referral to MTB
b. Broad sequencing versus unsolicited genetic findings
c. Clinical benefit for patients versus concern about expenses of diagnostics
d. MTB is indispensable versus currently only limited clinical significance
e. Hope versus disappointment about MTB advice
f. Off-label use versus evidence-based medicine
II. The organisation and regulation of an MTB
a. differences in main focus of MTB: lung oncology versus medical oncology versus research focus
b. stronger regional collaboration versus scarcity of time to participate
c. importance of evaluating efficacy of an MTB versus lack of time and resources to perform evaluation
III. Financial and logistical resources
a. enthusiasm about technological advances versus concern about increasing costs
b. reimbursement of the molecular test only versus including innovation costs in reimbursement
c. logistical support for MTB needed versus concern about increasing costs
d. experts prefer presence of referring clinician versus insufficient time of clinicians to attend MTB
e. health insurance companies might reimburse if outcomes are good and quality control is arranged versus currently no collection of outcomes and no quality requirements

## Discussion

Most clinicians value the MTB, primarily for the possible extra treatment options it provides for their patients. To improve MTBs, first, an easily accessible national or international registry is needed that collects molecular, treatment and follow-up data. Second, guidelines are needed concerning the molecular diagnostic methods, the provided clinical information, and the composition and organisation of the MTB. Third, financial arrangements for off-label drugs are a prerequisite to enable financial sustainability. Finally, regional collaboration is crucial in order to assure accessibility to an MTB for every eligible cancer patient. The large variety we observed in MTBs is in agreement with other studies.^1,2^ Several studies support evidence for the benefits of precision medicine.^3–7^ The first randomised study, the SHIVA trial, comparing precision medicine versus conventional therapy, was negative, although this study has considerable limitations. In the Netherlands, the DRUP study (NCT02925234), and in the United States the TAPUR study (NCT0269353), examine the efficacy of targeted agents based on specific mutations and correlate treatment outcome with data on whole gene sequencing. Furthermore, experiences with MTBs have been described by several other centres.^8–12^ Our study included five out of seven MTBs in the Netherlands; it would be of interest to extend this research to other countries. We included representatives of the patient federation. Yet, we acknowledge the difference in education concerning current developments in health care compared to many other patients.

In conclusion, a well-designed MTB contributes to more extensive personalised treatment options for patients, and furthermore, guides clinicians in their decision-making based on ongoing biotechnological advances. For financial reimbursement of MTB-guided care by insurance companies and governmental organisations, the implementation of quality guidelines and evidence of the clinical benefit of MTB-guided treatment are crucial.

## Supplementary information

Supplementary material

## Data Availability

The data generated and analysed for the current investigation are not publicly available but are available at from the corresponding author on reasonable request.
