# Supplementary material for: Molecular tumour boards and molecular diagnostics for patients with cancer in the Netherlands: experiences, challenges, and aspirations
Source: Br J Cancer. 2019 May 27;121(1):34–6. doi: 10.1038/s41416-019-0489-3 (PMC6738039; doi:10.1038/s41416-019-0489-3)
Supplement: Supplementary file 1 — Supplementary material [file 41416_2019_489_MOESM1_ESM.docx]

**Supplementary material**

**Molecular tumor boards and molecular diagnostics for patients with cancer in the Netherlands: experiences, challenges, and aspirations**

Annelieke E.C.A.B. Willemsen^1^ *, Sarah Krausz^2*^*, Marjolijn J.L. Ligtenberg^3,4^, Katrien Grünberg^3^, Harry J.M. Groen^5^, Emile E. Voest^6^, Edwin P.J.G. Cuppen^7,8^, Hanneke W.M. van Laarhoven^2^**, and Carla M.L. van Herpen^1^**

* these authors contributed equally to this work

** these authors contributed equally to this work

**Affiliations**

^1^ Department of Medical Oncology, Radboud university medical center, Nijmegen, The Netherlands

^2^ Department of Medical Oncology, Cancer Center Amsterdam, Amsterdam University Medical Centers, University of Amsterdam, The Netherlands

^3^ Department of Pathology, Radboud university medical center, Nijmegen, The Netherlands

^4^ Department of Human Genetics, Radboud university medical center, Nijmegen, The Netherlands

^5^ Department of Pulmonary Diseases, University Medical Center Groningen, Groningen, The Netherlands

^6^ Division of Molecular Oncology, Netherlands Cancer Institute, Amsterdam, The Netherlands

^7^ Center for Molecular Medicine and Oncode Institute, University Medical Center Utrecht, Utrecht, The Netherlands

^8^ Hartwig Medical Foundation, Amsterdam, The Netherlands

**Materials and methods**

**Data collection**

AW and SK, both medical oncologists in training at the time of study, carried out semi-structured interviews. They had no prior relationship with the participants. Interviews were held face-to-face, on the phone, or using a video call, and lasted approximately 30–60 min. The interview guide was developed based on key topics from literature and investigators’ knowledge and experiences from clinical practice and was further adapted in the course of the investigation. Interviews were audio recorded.

**Data analysis**

Interviews were transcribed and coded based on template analysis [^8^](#_ENREF_8). Template analysis is a thematic analysis where the researcher identifies a number of codes or themes that summarize key ideas, concepts or experiences extracted from the interviews by reading and re-reading the text. Codes are organized hierarchically with the highest level codes representing broad themes in the data and lower-level codes representing more narrow or specified themes in the data (Supplementary material, table 2). SK and AW analyzed the first two interviews for main codes, sub-codes and specific points of tension. A point of tension is an experience or opinion that is in disagreement with other respondents. In this case, a deeper interrogation was done in order to gain more insight. All other interviews were analyzed in a similar fashion and, where applicable, new codes and sub-codes were identified. All interviews were transcribed and reviewed for the final template. The template was further developed in discussion with HvL and CvH. AW and SK constructed the final template on the basis of detailed re-reading of the full set of transcripts and discussed their interpretations with CvH and HvL.

In the first stage, we interviewed 21 health professionals participating in an MTB (MTB-center), including at least a clinical scientist in molecular pathology and/or a pathologist, a medical oncologist and a lung oncologist. When possible, a clinical geneticist and/or a financial manager were interviewed as well. In the second stage, 15 health professionals were interviewed from similar disciplines as in stage one and working in a medical center without an MTB (non-MTB center). In the third stage, the following stakeholders were interviewed: three representatives of two health insurance companies, a group of circa 50 medical oncologists in training, and three representatives of the national cancer patient federation. The term ‘clinicians’ is used in case something applies for both medical oncologists and lung oncologists.

| **Table 1 Template analysis** | | | | |  |  |  |
| --- | --- | --- | --- | --- | --- | --- | --- |
| **1. Significance of MTB** | | | | |  |  |  |
|  | 1.1 perspective of patients | |  |  |  |  |  |
|  |  | 1.1.1. favor MTB advice for all oncological patients | | |  |  |  |
|  |  | 1.1.2. satisfied about extra therapeutic options | | |  |  |  |
|  |  | 1.1.3. satisfied about enrollment in early-stage clinical trials | | | |  |  |
|  |  | 1.1.4. off-label prescription courageous from physician | | |  |  |  |
|  |  | 1.1.5. favor early referral to MTB | |  |  |  |  |
|  |  | 1.1.6. policy on genetic unsolicited findings | | |  |  |  |
|  |  |  | 1.1.6.1. informing patients about genetic unsolicited findings is important and needed | | | | |
|  |  |  | 1.1.6.2. informing patients about genetic unsolicited findings can be confusing | | | | |
|  |  |  |  | 1.1.6.2.1. decision making tool for patients is helpful | | |  |
|  |  |  | 1.1.6.3. official informed consent is needed prior to every genetic test | | | |  |
|  |  |  | 1.1.6.4. opt in/opt out procedure is sufficient | | |  |  |
|  |  |  | 1.1.6.5. in case of genetic unsolicited findings a referral to a clinical geneticists is needed | | | | |
|  | 1.2 perspective of health professionals and other stakeholders | | | |  |  |  |
|  |  | 1.2.1. clinical significance of MTB | |  |  |  |  |
|  |  |  | 1.2.1.1. MTB is indispensable for physicians | | |  |  |
|  |  |  | 1.2.1.2. doubtful about current significance of MTB | | |  |  |
|  |  |  | 1.2.1.3. critical appraisal of (external) sequencing results | | | |  |
|  |  |  | 1.2.1.4. centralizing off-label prescriptions | | |  |  |
|  |  |  | 1.2.1.5. off-label prescriptions in some (rare) tumors indispensible | | | |  |
|  |  |  | 1.2.1.6. concern about lack of evidence in case of off-label use | | | |  |
|  |  |  | 1.2.1.7. off-label prescription undesirable | | |  |  |
|  |  | 1.2.2. influence MTB on patients' health care | | |  |  |  |
|  |  |  | 1.2.2.1. disappointment in case of no actionable mutation | | | |  |
|  |  |  | 1.2.2.1. delaying palliative care while waiting for result | | |  |  |
|  |  | 1.2.3. education and science | |  |  |  |  |
|  |  |  | 1.2.3.1. MTB-app to share eligible clinical trials | | |  |  |
|  |  |  | 1.2.3.2. sharing knowledge on biomedical and pharmaceutical issues | | | |  |
|  |  |  | 1.2.3.3. education of health care professionals (in training) | | | |  |
|  |  |  | 1.2.3.4. interdisciplinary knowledge transfer | | |  |  |
|  |  |  | 2.3.3.5. encourage scientific knowledge and output | | |  |  |
| **2. Organization and regulation of an MTB within a network** | | | |  |  |  |  |
|  | 2.1 interdisciplinary collaboration within an MTB | | |  |  |  |  |
|  |  | 2.1.1. satisfaction with interdisciplinary collaboration | | |  |  |  |
|  |  | 2.1.2. satisfaction with participation and preparation | | |  |  |  |
|  |  | 2.1.3. agreement on local organization and main goal of MTB | | | |  |  |
|  |  | 2.1.4. variety on case histories between different MTBs | | |  |  |  |
|  | 2.2 regional collaboration of MTB within their network | | |  |  |  |  |
|  |  | 2.2.1. more regional collaboration in a network needed | | | |  |  |
|  |  | 2.2.2. time is scarce to participate to MTB (non-MTB and MTB center) | | | |  |  |
|  |  | 2.2.3. wish to increase patient number for MTB | | |  |  |  |
|  |  | 2.2.4. attendance at tele-/videoconference to participate with MTB | | | |  |  |
|  | 2.3 national organization of MTB | |  |  |  |  |  |
|  |  | 2.3.1. discrepancy on local and national level | | |  |  |  |
|  |  |  | 2.3.1. collaboration of medical and lung oncologists on local level | | | |  |
|  |  |  | 2.3.2. no collaboration lung oncology and medical oncology medical associations | | |  |  |
|  |  | 2.3.2. future perspective | |  |  |  |  |
|  |  |  | 2.3.2.1. a non-tumor specific MTB in every academic center/specialized cancer institute | | | | |
|  |  |  | 2.3.2.2. one central MTB for high complex cases | | |  |  |
|  |  |  | 2.3.2.3. a tumor-specific MTB per expertise center | | |  |  |
|  |  |  | 2.3.2.4. MTBs eventually fuse in the tumor-specific working groups | | | |  |
|  | 2.4 quality control of an MTB | |  |  |  |  |  |
|  |  | 2.3.1. evaluation of functioning of MTB is essential | | | |  |  |
|  |  | 2.3.2. sufficient time and financial resources for evaluation are lacking | | | |  |  |
|  |  | 2.3.3. (re)define goals of MTB | |  |  |  |  |
|  |  | 2.3.4. discuss mutual expectations | |  |  |  |  |
|  |  | 2.3.5. setting up a registry for molecular/clinical data | | |  |  |  |
| **3. Financial and logistical resources** | |  |  |  |  |  |  |
|  | 3.1. costs of molecular diagnostic tests | | |  |  |  |  |
|  |  | 3.1.1. predictive diagnostic saves money | | |  |  |  |
|  |  | 3.1.2. provide evidence of (cost-)efficiency | | |  |  |  |
|  | 3.2 research and development costs of molecular diagnostic tests | | | |  |  |  |
|  |  | 3.2.1. costs for research and development included in sequencing costs | | | |  |  |
|  |  | 3.2.2. costs for research and development as separate funding only in academic center | | | | |  |
|  |  | 3.2.3. costs shared by other stakeholders: pharmaceutical companies, health insurance companies, hospitals | | | | | |
|  | 3.3 concern about costs of molecular diagnostic tests | | |  |  |  |  |
|  |  | 3.3.1. no concern about these costs: small part of total costs of oncological patient | | | | |  |
|  |  | 3.3.2. concern about rising costs of molecular diagnostic tests and about its relevance | | | | |  |
|  | 3.4 immaterial costs | |  |  |  |  |  |
|  |  | 3.4.1. time expenses for MTB participants in case of a treatment advice without patient referral to MTB center | | | | | |
|  |  |  | 4.5.2.1. no financial compensation for MTB clinician | | |  |  |
|  |  |  | 4.5.2.2. financial compensation for MTB clinician | | | |  |
|  |  | 3.4.2. time expenses for non-MTB clinicians | | |  |  |  |
|  |  |  | 4.5.3.1. concern about time expenses for MTB | | |  |  |
|  |  |  | 4.5.3.2. time expenses for MTB included in daily work | | |  |  |
|  | 3.5. logistical aspect**s** | |  |  |  |  |  |
|  |  | 3.5.1. support and facilitation should be improved | | |  |  |  |
|  |  |  | 3.5.1.1. managing the enrollment of patients | | |  |  |
|  |  |  | 3.5.1.2. managing the agenda of the meeting | | |  |  |
|  |  |  | 3.5.1.3. managing the patient reports | | |  |  |
|  |  |  | 3.5.1.4. enrollment of patients via a website | | |  |  |
|  | 3.6. patient report MTB | |  |  |  |  |  |
|  |  | 3.6.1. molecular test result with a clear conclusion and treatment recommendation | | | | |  |
|  |  | 3.6.2. description of the used molecular test or method | | |  |  |  |
|  |  |  |  |  |  |  |  |

**Table 2 Respondent characteristics**

| **Medical center** | **Medical center** | **MTB center** | **Respondent** | **Gender** | **Profession** | **Type of interview** |
| --- | --- | --- | --- | --- | --- | --- |
| 1 | non-academic* | yes | 1 | F | CSMP | by telephone |
|  |  |  | 2 | M | Pa | by telephone |
|  |  |  | 3 | M | MO | by telephone |
|  |  |  | 4 | M | LO | by telephone |
| 2 | academic | yes | 5 | M | CSMP | by telephone |
|  |  |  | 6 | M | MO | by telephone |
|  |  |  | 7 | M | LO | by telephone |
| 3 | academic | yes | 8 | F | CSMP | in the hospital |
|  |  |  | 9 | M | MO | in the hospital |
|  |  |  | 10 | F | CG | in the hospital |
| 4 | academic | yes | 11 | M | CSMP | by telephone |
|  |  |  | 12 | M | Pa | by telephone |
|  |  |  | 13 | F | MO | by telephone |
|  |  |  | 14 | M | LO | by telephone |
| 5 | academic | yes | 15 | F | CSMP | in the hospital |
|  |  |  | 16 | F | Pa | in the hospital |
|  |  |  | 17 | F | MO1 | in the hospital |
|  |  |  | 18 | M | MO2 | in the hospital |
|  |  |  | 19 | M | LO | in the hospital |
|  |  |  | 20 | F | CG | in the hospital |
|  |  |  | 21 | M | FM | in the hospital |
| 6 | non-academic | no | 22 | F | CSMP | by telephone |
|  |  |  | 23 | M | MO | in the hospital |
|  |  |  | 24 | F | LO | by telephone |
|  |  |  | 25 | M | FM | in the hospital |
| 7 | academic | no | 26 | F | MO | video-call |
|  |  |  | 27 | M | SU | by telephone |
|  |  |  | 28 | M | Pa | by telephone |
|  |  |  | 29 | M | CSMP | by telephone |
| 8 | non-academic | no | 30 | F | MO | by telephone |
|  |  |  | 31 | M | CSMP | by telephone |
|  |  |  | 32 | F | LO | by telephone |
| 9 | non-academic | no | 33 | F | MO | by telephone |
|  |  |  | 34 | M | FM | by telephone |
| 10 | non-academic | no | 35 | M | MO | by telephone |
|  |  |  | 36 | F | LO | by telephone |
| na | na | na | multiple | na | Patient federation | live or by e-mail |
|  |  |  | multiple | na | Health insurance company | live |
|  |  |  | multiple | na | Junior oncologists | live |

* specialized cancer center defined as academic center

M= male, F= female

CSMP = clinical scientist in molecular pathology

Pa = Pathologist

MO = medical oncologist

LO = lung oncologist

CG = clinical geneticist

FM = financial manager

SU = surgeon

na= not applicable
